# Comparison of Involved Field Radiotherapy versus Elective Nodal Irradiation in Stage IIIB/C Non-Small-Cell Lung Carcinoma Patients Treated with Concurrent Chemoradiotherapy: A Propensity Score Matching Study

**DOI:** 10.1155/2020/7083149

**Published:** 2020-09-04

**Authors:** Erkan Topkan, Yurday Ozdemir, Ozan Cem Guler, Ahmet Kucuk, Ali Ayberk Besen, Huseyin Mertsoylu, Duygu Sezen, Eyub Yasar Akdemir, Ahmet Sezer, Yasemin Bolukbasi, Berrin Pehlivan, Ugur Selek

**Affiliations:** ^1^Baskent University Medical Faculty, Department of Radiation Oncology, Adana, Turkey; ^2^Mersin City Hospital, Radiation Oncology Clinics, Mersin, Turkey; ^3^Baskent University Medical Faculty, Department of Medical Oncology, Adana, Turkey; ^4^Koc University, School of Medicine, Radiation Oncology Department, Istanbul, Turkey; ^5^Bahcesehir University, Department of Radiation Oncology, Istanbul, Turkey; ^6^U. T. MD Anderson Cancer Center, Radiation Oncology Department, Houston, TX, USA

## Abstract

**Background:**

We retrospectively compared the incidence of isolated elective nodal failure (IENF) and toxicity rates and survival outcomes after elective nodal irradiation (ENI) versus involved-field RT (IFRT) by employing the propensity score matching (PSM) methodology in stage IIIB/C inoperable non-small-cell lung cancer (NSCLC) patients treated with definitive concurrent chemoradiotherapy (C-CRT).

**Methods:**

Our PSM examination included 1048 stage IIIB/C NSCLC patients treated with C-CRT from January 2007 to December 2016: a total dose of 66 Gy (2 Gy/fraction) radiotherapy and 1–3 cycles of platinum-based doublet chemotherapy concurrently. The primary and secondary endpoints were the IENF and toxicity rates and survival outcomes after ENI versus IFRT, respectively. Propensity scores were calculated for each group to adjust for confounding variables and facilitate well-balanced comparability by creating 1 : 1 matched study groups.

**Results:**

The median follow-up was 26.4 months for the whole study accomplice. The PSM analysis unveiled 1 : 1 matched 646 patients for the ENI (*N* = 323) and IFRT (*N* = 323) cohorts. Intergroup comparisons discovered that the 5-year isolated ENF incidence rates (3.4% versus 4.3%; *P*=0.52) and median overall survival (25.2 versus 24.6 months; *P*=0.69), locoregional progression-free survival (15.3 versus 15.1 months; *P*=0.52), and progression-free survival (11.7 versus 11.2 months; *P*=0.57) durations were similar between the ENI and IFRT cohorts, separately. However, acute grade 3-4 leukopenia (*P*=0.0012), grade 3 nausea-vomiting (*P*=0.006), esophagitis (*P*=0.003), pneumonitis (*P*=0.002), late grade 3-4 esophageal toxicity (*P*=0.038), and the need for hospitalization (*P* < 0.001) were all significantly higher in the ENI than in the IFRT group, respectively.

**Conclusion:**

Results of the present large-scale PSM cohort established the absence of meaningful IENF or survival differences between the IFRT and ENI cohorts and, consequently, counseled the IFRT as the elected RT technique for such patients since ENI increased the toxicity rates.

## 1. Introduction

Platinum-based concurrent chemoradiotherapy (C-CRT) represents the current care standard for the medically fit but unresectable stage III non-small-cell lung carcinoma (NSCLC) patients [1, 2]. Traditionally, the radiotherapy (RT) portal encompasses the index lung primary plus the mediastinal and ipsilateral hilar lymph nodes (LNs), and not infrequently the supraclavicular LNs, negligent of their involvement status: elective nodal irradiation (ENI) technique [3]. However, aggressive C-CRT protocols with ENI failed to improve the prognosis of stage IIIB/C patients, and indeed, such large RT portals have been proven to assuredly increase the rates of acute toxicities, especially the acute esophagitis and pneumonitis, and diminish the tolerability of prescribed C-CRT regimes [4, 5].

Opposed to the ENI, involved field RT (IFRT) permits feasible escalation of the RT doses to involved areas with notably decreased doses received by the dose-limiting healthy critical structures via exclusive inclusion of the primary tumor site and involved LNs in the RT portal [6–11]. Hypothetically, IFRT may meaningfully improve the therapeutic index by positively enhancing the clinical outcomes and reducing the acute and late complication rates. Nevertheless, conversely, the 25% false-negative rates in PET-CT staged <1 cm LNs and postoperative 10–35% rates of occult LN metastases in clinical stage I NSCLC patients collectively suggest increased risks for nodal failures with IFRT, as the clinically uninvolved LN stations are not irradiated [12–16]. But, starkly contradicting with such evidence, the scarce IFRT studies and a meta-analysis successfully showed that the isolated elective nodal failures (IENFs) after IFRT were consistently less than 10%, essentially when PET-CT was utilized as the initial staging tool [6–11].

To date, only relatively small-scale studies compared the IFRT and ENI in terms of IENF and clinical outcomes, and even the largest randomized controlled trial by Yuan et al. randomized only 100 patients to either treatment arms [10]. Moreover, to the best of our knowledge, the propensity score matching (PSM) analysis, which can efficiently adjust for confounders and facilitate a well-balanced comparison between the two retrospectively analyzed patients groups, has never been used in this context before. Hence, this retrospective analysis in 1048 stage IIIB/C NSCLC patients treated with definitive C-CRT endeavored to determine the incidences of IENF after ENI and IFRT and objectively compare these two RT techniques in terms of acute toxicity rates and survival outcomes by utilizing the PSM methodology.

## 2. Patients and Methods

### 2.1. Study Population

The institutional database kept by the Baskent University Medical Faculty Department of Radiation Oncology was retrospectively sought to identify all stage IIIB/CNSCLC patients who had undergone C-CRT between January 2007 and December 2016. Inclusion criteria were as follows: age between 18 and 80 years, Karnofsky Performance Score (KPS) ≥ 70, histopathologically proven adenocarcinoma (AC) or squamous cell carcinoma, stage IIIB/C per AJCC 8^th^ ed., body mass index ≥ 18.5 kg/m^2^, accessible pretreatment brain magnetic resonance imaging (MRI) scans, treatment charts and hospital computerized treatment data sets, no prior history of thoracic RT/chemotherapy, and to be undergone at least 1 cycle of concurrent chemotherapy during the course of a total dose of 66 Gy (2 Gy per daily fractions) thoracic RT course. Pretreatment evidence for any of the following factors is considered as exclusion criteria: inadequate pulmonary, cardiac, renal, or hepatic functions, inadequate blood count/chemistry tests, and presence of any of the contralateral supraclavicular LN involvement and/or malignant pleural/pericardial effusions.

The study design was approved by the institutional review board of the Baskent University Medical Faculty before any patient data acquisition, and written informed consent was provided by each participant by either themselves or legally authorized representatives for collection and analysis of blood samples, pathologic specimens, and publication of the outcomes.

### 2.2. Concurrent Chemoradiotherapy

All RT plans were typically accomplished by employing coregistered diagnostic CT and PET fusion scans as per our institutional care standard for likely LA-NSCLC patients as of January 2007. Patients were treated with 3-dimensional conformal RT (3D-CRT) or intensity-modulated RT (IMRT). Target volume definition and dose specifications and organ at risk dose limits were as formerly portrayed by Topkan and colleagues [17], elsewhere. In brief, 66 Gy in 2 Gy per fraction was standardly endorsed to all patients regardless of the RT procedure. Simultaneous integrated boost IMRT, split course RT, and induction chemotherapy were not allowed. Every qualified patient received 1 to 3 cycles of cisplatin/carboplatin plus one of docetaxel/paclitaxel (taxanes), vinorelbine, or etoposide concurrent with RT. Regular supportive and symptomatic care measures were offered as indicated precisely.

### 2.3. Volume Definition and Contouring

Gross tumor volume (GTV) was defined as the apparent extent of the primary tumor and the metastatic LNs, respectively. Involved nodal GTV was defined as metabolically avid in PET or abnormally enlarged regional LNs measuring over 1.0 cm along their short axis in CT scans. CTV for IFRT was GTV plus 6 and 8 mm margins for SCC and AC histologies, respectively [18]. CTV was restrained to avoid extending beyond anatomic boundaries (chest wall, vertebral body, great vessels, heart, esophagus, etc.) except for verifiable sufficient evidence for the invasion. CTV for ENI is needed to cover related mediastinum ± ipsilateral supraclavicular LNs (if primary located in upper lobes and main stem bronchus) and ipsilateral hilum, even in the absence of clinical or radiological proof of involvement [19].

### 2.4. Toxicity and Response Assessment

Each eligible patient underwent routine toxicity evaluations at weekly intervals or more often during the C-CRT, as indicated. Common Terminology Criteria for Adverse Events v3 scoring criteria were used for acute and late toxicity assessments, and the reported scores mirrored the worst grade discerned. After the completion of the C-CRT, patients were assessed every 3 months for the first 2 years, 6 months for the 3 to 5 years, and then yearly or more frequently whenever demanded.

Treatment response was surveyed by restaging PET-CT scans starting from the 3-month post-C-CRT follow-up per the PET Response Criteria in Solid Tumors (PERCISTs). Next, all patients were monitored by the blood counts/chemistry tests and PET-CT or chest CT (whenever metabolic complete response is confirmed) at interims meant above. Additional abdominal ultrasound and/or CT, PET-CT, and bone scintigraphy were carried out for restaging if necessitated, while cranial MRI was utilized only if clinically wavered.

### 2.5. Statistical Analyses

Our primary endpoint was to compare the IENF rates between the ENI and IFRT cohorts, while the respective comparative assessment of the acute and late toxic events, overall survival (OS: interval between the first day of C-CRT and death/last visit), locoregional progression-free survival (LRPFS: interval between the first day of C-CRT and recurrence or progression at the primary tumor site and/or ipsi- and/or contralateral hilum/mediastinum or death), and PFS (interval between the first day of C-CRT and any type of disease progression on last visit or death) between the two treatment groups comprised the secondary endpoints. The IENF was described as any nodal failure beyond the 95% isodose line covering the intended PTV for IFRT technique, while any nodal recurrences excluding the initially involved ones, to be specific the GTV lymph nodes, were accepted as IENF for the ENI technique irrespective of their status of whether being covered with the PTV or not.

Frequency distributions were used to describe categorical variables, and the Chi-square test, Student's *t*-test, Pearson's exact test, or Spearman's correlation estimates were used to compare their differences between the groups, appropriately. Medians and ranges were employed to express quantitative variables. Patients were categorized into the required number of groups for statistical comparisons when necessitated. The respective Kaplan-Meier and log-rank tests were performed to compare the outcomes per the potential risk factor. The Cox Proportional Hazards model was operated for the multivariate comparisons by holding only the variables manifesting significance in univariate comparisons. All listed *P* values were two-sided, and *P* < 0.05 was deemed significant.

Because the present research was a large-scale retrospective cohort examination, we calculated the propensity scores for each of IENF and toxicity rates and survival outcomes to adjust to the likely confounding factors and to facilitate well-balanced comparability between the ENI and IFRT cohorts. In this manner, we generated 1 : 1 matched cohorts with a caliper width of 0.05.

## 3. Results

Our database search identified a sum of 1572 stage IIIB/C NSCLC patients. Nevertheless, 1048 of them were eligible for the current examination; 524 of them were decided to be incompetent due to receiving induction chemotherapy before the C-CRT (*N* = 251), <66 Gy RT (*N* = 106), hypofractionated RT (*N* = 86), and RT alone (*N* = 81). Among the eligible 1048 patients, 379 (36.2%) received ENI, while the remaining 669 (63.8%) patients underwent IFRT. Patients and treatment characteristics for the entire study population and per RT technique (ENI versus IFRT) were as displayed in Table 1. Even though the baseline characteristics were comparably distributed within the ENI and IFRT gatherings, the ability to receive the 2-3 cycles of the designated doublet chemotherapy was significantly higher in the IFRT than the ENI group (87.0% versus 71.8%; *P*=0.002).

A total of 646 patients out of 1048 were 1 : 1 group-matched with 323 patients on each of the ENI and IFRT gatherings as per the PSM methodology. The patient and disease characteristics stayed to be evenly dispersed with no remarkable distinction between the two cohorts after the PSM grouping (Table 2). The results introduced hereinafter will signify the outcomes of PSM cohorts, if not stipulated otherwise.

The C-CRT was relatively well tolerated by the entire PSM cohort with no report of grades 4-5 acute nonhematologic and grade 5 hematologic toxicities (Table 3). Grade 3-4 leukopenia (15.7% versus 9.9%; hazard ratio (HR):1.64; *P*=0.012), nausea-vomiting (33.7% versus 20.7%; HR: 1.73; *P*=0.006), esophagitis (15.2% versus 9.0%; HR: 1.77; *P*=0.003), pneumonitis (14.5% versus 8.4%; HR: 1.69; *P*=0.002), and obligation for hospitalization because of severe acute toxic events (18.0% versus 8.6%; HR: 2.17; *P* < 0.001) were altogether significantly higher in the ENI than the IFRT cohort. Late grade 3-4 esophagitis was likewise significantly more frequent in the ENI group (4.6% versus 2.5%; HR: 1.91; *P*=0.038). Overall, there were 10 (1.6%) late grade 5 events (*N* = 6 (1.8%) in ENI versus *N* = 4 (1.2%) in IFRT; *P*=0.73), as specified in Table 3. Of those, barring the 3 deaths associated with radiation pneumonitis, the progressive disease may likewise be related to mortality in the remaining 7, as there were simultaneous signs of disease progression in these cases.

The IENF was diagnosed in 25 (3.9%) and 35 (5.4%) patients at 5- and 10-year time points in the whole PSM cohort, with no statistically meaningful difference between the ENI or IFRT cohorts at either of the 5- (3.4% versus 4.3%; *P*=0.52) or 10-year (4.9% versus 5.9%; *P*=0.71) endpoints (Table 3).

**Figure 1:**
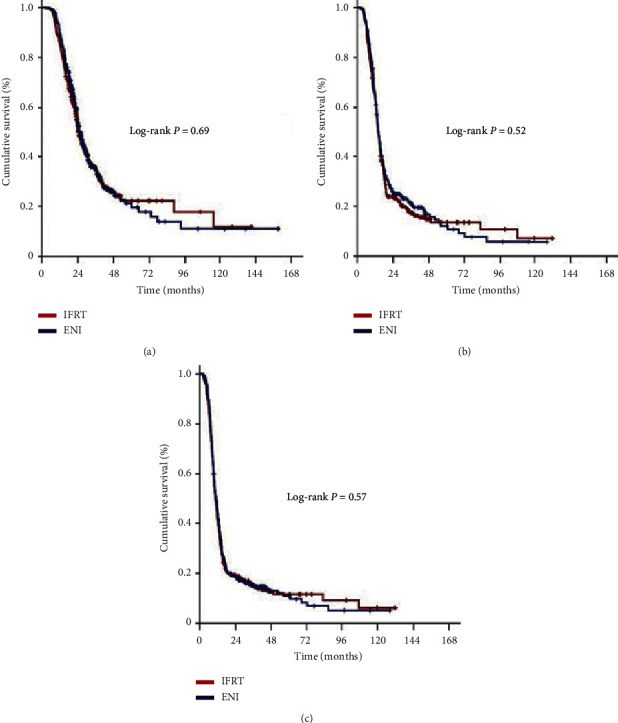
Results of comparative survival analysis according to the elective nodal irradiation (ENI) versus involved-field radiotherapy (IFRT) status: (a) overall survival, (b) locoregional progression-free survival, and (c) progression-free survival.

The median follow-up was 26.4 months (95% confidence interval (CI): 19.8–33.0) during the final analysis for the whole PSM assembly. Of these patients, 236 (36.5%) were still alive: 115 (35.6%) and 121 (37.5%) in the ENI and IFRT cohorts, respectively (*P*=0.84); and 87 (13.5%) were free of disease progression: 45 (13.9%) and 42 (13.0%) in the ENI and IFRT cohorts, respectively (*P*=0.91). For the whole PSM gathering, the median, 5-year, 10-year OS, LRPFS, and PFS estimates were 24.9 months (95% CI: 23.5–26.3), 20.8%, and 11.5%, 15.2 months (95% CI: 14.5–15.9), 12.8%, and 6.4%, and 11.4 months (95% CI: 10.8–12.0), 11.3%, and 5.6%, individually (Table 3). Comparative analyses between the ENI and IFRT cohorts exhibited that the median OS (25.2 versus 24.6 months; *P*=0.69), LRPFS (15.3 versus 15.1 months; *P*=0.52), and PFS (11.7 versus 11.2 months; *P*=0.57) durations were statistically indistinguishable, separately (Figure 1). Further, as depicted in Table 3 and Figure 1, the long-term survival results at 5- and 10-year time points did not prove any significant separation in Kaplan-Meier curves for any of the OS, LRPS, or PFS outcomes in favor of ENI or IFRT companions.

In univariate analysis, by entering the variables portrayed in Table 1, we discovered that the KPS 90–100 (versus 70–80), T1-2 stage (versus T3-4), N2 stage (versus N3), overall disease stage IIIB (versus IIIC), and ability to receive 2-3 cycles of prescribed chemotherapy (versus 1 cycle) were the factors manifesting a statistically significant link with each of the respective OS, LRPFS, and PFS outcomes (Table 4). Restricted to these factors, the results of the multivariate analyses unveiled that all factors were independently associated with superior outcomes with regard to all survival endpoints (Table 4).

## 4. Discussion

This retrospective single institutional, large-scale PSM analysis was performed to compare the incidences of IENF, toxic events, and survival outcomes between the IFRT and ENI in stage IIIB/C NSCLC patients managed with platinum-based C-CRT. We could not reveal any significant outcome benefit but significantly increased acute and late side effects for ENI over IFRT in our retrospective cohort, which to the best of our knowledge is the largest consistently staged and treated single-center data using PET-CT in the initial staging, RT planning, and follow-up response assessment phases, in the absence of notable discordances among the RT doses of the two groups.

Post-RT local failures have been reported to be remarkably more frequent than IENFs in retrospective and prospective ENI series [20, 21]. Sanuki-Fujimoto et al. retrospectively examined the noteworthiness of treating clinically uninvolved LNs in a cohort of 127 patients who received conventionally fractionated 60 to 68 Gy to the primary tumor plus the involved LNs and electively 40 Gy to the uninvolved subclinical regions [20]. The researchers reported no IENF, with in-field local/regional and distant failures being the leading failure types at a median follow-up time of 50.5 months. Emami et al., on the other hand, analyzed 1705 unresectable NSCLC patients from four large-scale RTOG trials (78-11, 79-17, 83-11, and 84-07) for the impact of nodal RT on regional progression and survival [21]. Although the authors underscored the importance of ipsilateral hilar coverage to prevent the in-field progression (11.6 vs. 22% for adequately vs. inadequately covered ipsilateral hilum, respectively; *P*=0.01), proposing the inutility of ENI, this distinction did not translate into a meaningful survival benefit at 2 years (35% vs. 37%; *P* > 0.05). Like the RTOG researchers, we also found that the major failure pattern was not a regional failure in nontargeted fields, but local/regional in initially irradiated volume, if not distant metastasis.

The quantity of the incidental dose, elective nodal coverage, and its adequacy for the microscopic disease control was not tended in our PSM cohorts; however, these issues were addressed by Polish and Japanese retrospective studies [22, 23]. Kepka et al. estimated the incidental doses and *V*_40_ values of 3D-CRT for specific LN stations with varying ENI extents (extended, limited, and omitted) in 220NSCLC patients [22]. They have noticed that the incidental irradiation of unintended LNs was apparent in case of limited ENI, such as uninvolved stations of 5 and 6, and lower parts of 3A and 3P were already irradiated while electively irradiating stations of 4R, 4L, 7, and ipsilateral hilum, but these stations were not adequately covered in IFRT, and levels 1 and 2 were not covered in any scenario. Then again, Kimura et al. assessed the incidental ENI in 50 advanced NSCLC patients undergoing IFRT and proclaimed that the low incidence of IENF might be related to significant unintended ENI doses in patients receiving IFRT by revealing that most of the ENI regions, except levels 1, 3, and 7, were still exposed to significant doses despite being not targeted deliberately [23]. Although we did not portray the uninvolved nodal levels in our IFRT gathering, yet, like the previous IFRT research, it sounds rational to predict that the adjacent nodal areas might have received relatively small but radiobiologically significant incidental ENI doses in some patients.

Rosenzweig et al. published the first admirable effort on IFRT in a cohort of 171 NSCLC patients treated with 3D-CRT between 1991 and 1998, where none of the elective LN regions were irradiated if not proved to be involved by either biopsy or radiographic criteria [6]. The authors reported only 11 (6.4%) IENFs at a median follow-up of 21 months. Later, the same group updated their dose-escalated IFRT results in 524 NSCLCs and, again, reported an IENF rate of only 6.1% at a median follow-up of 41 months [7]. Inline, Sulman et al. published their experience of IFRT in 115 NSCLCs managed with definitive RT or C-CRT and noted 4.3% ENFs with only 1.7% IENF rate [8]. Fernandes et al. retrospectively analyzed the results of 108 consecutive patients who underwent ENI (*N* = 60) or IFRT (*N* = 48) for stage III or stage IV oligometastatic NSCLCs [24]. No statistically significant distinction was reported in terms of 2-year local control (39.2% vs. 59.6%), elective nodal control (84.3% vs. 84.3%), distant control (47.7% vs. 52.7%), and OS (40.1% vs. 43.7%) between ENI versus IFRT cohorts. In our PSM accomplice of 646 patients with a median follow-up of 26.4 months, the traditional KPS, T-stage, N-stage, TNM stage, and the number of concurrent chemotherapy cycles were identified to exert independent prognostic worth on the OS, LRPFS, and PFS, but confirming the previous studies, we have not noticed any outcome difference between the ENI and IFRT cohorts in terms of OS (25.2 versus 24.6 months; *P*=0.69), LRPFS (15.3 versus 15.1 months; *P*=0.49), and PFS (11.7 versus 11.2 months; *P*=0.57) endpoints.

Because incidental ENI of 3D-CRT was recognized as a typical by-product of 3D-CRT, a hypothetically decreased incidental dose deposition in elective nodal stations with the efficient delivery of IMRT-based IFRT has been extensively discussed. Fleckenstein et al. established a planning study (41 patients, CTV: GTV + 3 mm, and all PET-positive LN stations; PTV: CTV + 7 mm, dose-escalated until the predefined normal tissues dose-constraints) comparing IMRT versus 3D-CRT to analyze the impact on dosimetric parameters, equivalent uniform dose (EUD), and tumor control probabilities (TCP) (for the microscopic disease, a *D*_50_ of 36.5 Gy was assumed) in elective nodal stations [25]. They were convincingly able to show significantly higher total PTV doses with IMRT (74.3 Gy versus 70.1 Gy; *P*=0.03) but significantly lower EUD in LN stations adjacent to the CTV with IMRT than with 3D-CRT (40.4 Gy vs. 44.2 Gy; *P*=0.05) and a significant reduction of TCP with IMRT versus 3D-CRT for all LN stations not included in the CTV (23.6% vs. 27.3%) [25]. IFRT with IMRT achieved significantly better sparing of normal tissues with a higher total target dose where there might be a potential therapeutic drawback of decreased incidental ENI doses. Having said that, 3D-CRT in our PSM cohort was utilized in 38.4% patients (ENI, 39.3% vs. IFRT, 37.5%; *P*=0.69), and we have not recalled any effect of the RT technique on recurrence patterns.

The phase II prospective RTOG 0515 trial was conducted to quantify the relative impacts of coregistered PET-CT versus CT on RT plans and to determine the corresponding rates of IENF in 47 evaluable patients [9]. Overall, only 1 (2%) patient has developed an ENF with no notable difference in the number of involved LNs (2.1 vs. 2.4), *V*_20_ lung dose (32% vs. 30.8%), or mean esophageal dose (28.7 vs. 27.1 Gy) at a median follow-up of 12.9 months. However, supporting the RTOG standard of constraining the target volume to the primary tumor and involved LNs, the PET/CT-derived nodal GTV volumes were smaller and altered contouring in 51% of patients with no significant influence on IENF. Kolodziejczyk et al. in their PET-CT staged retrospective cohort of 67 patients (17 with ENI and 50 with IFRT) found that the IENF was experienced by only 3 (6.0%) patients after IFRT [26]. Fleckenstein et al. later documented the PET-based target volume delineation with the IFRT concept for IMRT and concluded that IMRT with IFRT did not forfeit any endpoints [27]. We have indeed used PET-CT not only at the initial ideal staging but also in the primary tumor and nodal contouring in all patients and revealed relatively low IENF rates (ENI vs. IFRT: at 5 years, 3.4% vs. 4.3%, *P*=0.52; at 10 years, 4.9% vs. 5.9%, *P*=0.71) like the abovementioned studies.

The first prospective phase III randomized study was by Yuan et al. comparing the dose-escalated IFRT (68 to 74 Gy) and ENI (60 to 64 Gy) in 200 inoperable stage III NSCLCs treated with C-CRT [10]. The authors remarked superior 5-year local control (51% vs. 36%, *P*=0.032), lower radiation pneumonitis (17% vs. 29%, *P*=0.044), and longer 2-year survival rates (25.6% vs. 39.4%; *P*=0.048) with IFRT [10]. Nevertheless, their study could mainly be criticized for the suboptimal staging procedure without PET, insufficiently powered population size, higher dose in the IFRT arm, and the subjective definition of LRC. Consequently, Chen et al. enrolled 99 LA-NSCLC patients on their prospective randomized C-CRT study comparing IFRT and ENI [28]. Although the median RT dose was 60 Gy, 36 (87.8%) and 40 (80.0%) patients in IFRT and ENI arms received >60 Gy, respectively. Besides the interestingly improved LRPFS and OS rates with IFRT, as no increase in initially uninvolved nodal or IENF risks was observed, the authors proposed that a notable decrease in IENF rates might be realized due to the augmented local control rates with IFRT. Li et al. conducted a praiseworthy comprehensive meta-analysis to provide Level 1 evidence on the peculiar incidences of IENF in IFRT and ENI procedures [11]. The results of 3 randomized controlled trials (RCTs) and 3 cohort studies uncovered no eminent disparity in the incidence of IENF among the RCTs (RR = 1.38; *P*=0.46), cohort studies (RR = 0.99; *P*=0.97), or combined (RR = 1.15; *P*=0.64), which are completely in good concordance with our PSM analysis revealing no outcome difference between the ENI and IFRT techniques.

We essentially have defined a significant increase in acute (nausea-vomiting: 33.7% vs. 20.7%; *P*=0.006, esophagitis: 15.2% vs. 9.0%; *P*=0.003, pneumonitis: 14.5% vs. 8.4%; *P*=0.002) and late grade ≥ 3 nonhematologic toxicities (esophageal toxicity: 4.6% vs. 2.5%, *P*=0.038), acute grade 3-4 hematologic toxicity (leukopenia: 15.7% vs. 9.9%; *P*=0.012), and hospitalization (18% vs. 8.6%; *P* < 0.001) rates in case of ENI. This increased toxicity pattern was not affected by any indicator including the treatment technique (IMRT vs. 3D-CRT) in our series, except for being treated by ENI or not. These increased toxicities are consistent with the reported literature [24]. For example, Fernandes et al. pointed out the odds of developing high-grade esophagitis to be 3.2 times higher with ENI in comparison to IFRT [24]. Grills et al. documented a dosimetric prediction study comparing ENI to IFRT by 3D-CRT or IMRT and showed a higher mean esophagus *V*_50_ in the ENI group compared to the IFRT group (34% vs. 15–18%) [29].

Although our data is retrospective, we have performed PSM analysis to minimize the selection bias and the influence of potential confounders and imbalances. In addition, we have key strengths in comparison to previous reports in the literature, such as being the largest uniformly staged and treated single-center data, no discordance between the IFRT and ENI groups regarding the RT doses, use of PET-CT in both staging and planning procedures, adequate follow-up period and radiologically sound routine, and avoiding inhomogeneity via PSM analysis between the IFRT and ENI patients groups. Therefore, in the absence of a well-powered, randomized, homogeneously treated prospective phase III trial, we believe that our results are sound and constructive in the emerging literature.

## 5. Conclusion

Our retrospective PSM cohort provides reliable and stable evidence in concordance with the given literature that there is no significant outcome difference in any of the IENF or survival endpoints between the IFRT and ENI but increased toxicity rates with ENI.

## Figures and Tables

**Table 1:** Pretreatment and treatment characteristics for all patients and per elective nodal irradiation status.

Covariate	All patients (*N* = 1048)	ENI (*N* = 379)	IFRT (*N* = 669)	*P* value
Median age, y (range)	62 (29–79)	61 (29–79)	64 (34–79)	0.56

Age group, y (%)				
≤70 years	927 (88.5)	328 (86.5)	599 (89.5)	0.43
>70 years	121 (11.5)	51 (13.5)	70 (10.5)	

Gender (%)				
Male	654 (62.4)	235 (62.0)	419 (62.6)	0.73
Female	394 (37.6)	144 (38.0)	250 (37.4)	

KPS (%)				
90–100	751 (71.7)	269 (71.0)	482 (72.0)	0.64
70–80	297 (28.3)	110 (29.0)	187 (28.0)	

Histology (%)				
AC	567 (54.1)	212 (55.9)	355 (53.1)	0.39
SCC	481 (45.9)	167 (44.1)	314 (46.9)	

T-stage				
1-2	361 (34.4)	116 (30.6)	245 (36.6)	0.28
3-4	687 (65.6)	263 (69.4)	424 (63.4)	

N-stage				
2	531 (50.7)	195 (51.5)	336 (50.2)	0.55
3	517 (49.3)	184 (48.5)	333 (49.8)	

Tumor stage (%)				
IIIB	617 (58.9)	217 (57.3)	400 (59.8)	0.67
IIIC	431 (41.1)	162 (42.7)	269 (40.2)	

Chemotherapy cycles (%)				
2-3	854 (81.4)	272 (71.8)	582 (87.0)	0.002
1	194 (18.6)	107 (28.2)	87 (13.0)	

Radiotherapy technique (%)				
3D-CRT	385 (36.7)	157 (41.4)	228 (34.1)	0.24
IMRT	663 (63.3)	222 (58.6)	441 (65.9)	

ENI: elective nodal irradiation; IFRT: involved-field radiotherapy; KPS: Karnofsky performance score; AC: adenocarcinoma; SCC: squamous cell cancer; T: tumor; N: node; 3D-CRT: 3-dimensional conformal radiotherapy; IMRT: intensity-modulated radiotherapy.

**Table 2:** Pretreatment and treatment characteristics for propensity score-matched patients per elective nodal irradiation status.

Covariate	All patients (*N* = 646)	ENI (*N* = 323)	IFRT (*N* = 323)	*P* value
Median age, *y* (range)	63 (31–79)	63 (31–79)	64 (34–78)	0.92

Age group, *y* (%)				
≤70 years	586 (90.7)	294 (91.0)	292 (90.4)	0.86
70 years	60 (9.3)	29 (9.0)	31 (9.6)	

Gender (%)				
Male	400 (61.9)	201 (62.2)	199 (61.6)	0.81
Female	246 (38.1)	122 (37.8)	124 (38.4)	

KPS (%)				
90–100	461 (71.4)	232 (71.8)	229 (70.9)	0.74
70–80	185 (28.6)	91 (28.2)	94 (29.1)	

Histology (%)				
AC	348 (53.9)	177 (54.8)	171 (52.9)	0.62
SCC	298 (46.1)	146 (45.2)	152 (47.1)	

T-stage				
1-2	289 (44.8)	143 (44.3)	146 (45.2)	0.43
3-4	357 (55.2)	180 (55.7)	177 (54.8)	

N-stage				
2	325 (50.3)	164 (50.8)	161 (49.8)	0.79
3	321 (49.7)	159 (49.2)	162 (50.2)	

Disease stage (%)				
IIIB	385 (59.6)	195 (60.4)	190 (58.8)	0.57
IIIC	261 (40.4)	128 (39.6)	133 (41.2)	

Chemotherapy cycles (%)				
2-3	529 (81.9)	263 (81.4)	266 (82.3)	0.71
1	117 (18.1)	60 (18.6)	57 (17.7)	

Radiotherapy technique (%)				
3D-CRT	248 (38.4)	127 (39.3)	121 (37.5)	0.69
IMRT	398 (61.6)	196 (60.7)	202 (62.5)	

ENI: elective nodal irradiation; IFRT: involved-field radiotherapy; KPS: Karnofsky performance score; AC: adenocarcinoma; SCC: squamous cell cancer; T: tumor; N: node; 3D-CRT: 3-dimensional conformal radiotherapy; IMRT: intensity-modulated radiotherapy.

**Table 3:** Toxicity and survival outcomes according to elective nodal irradiation status.

Outcome	ENI (*N* = 323)	IFRT (*N* = 323)	*P* value
Acute grade 3-4 hematologic toxicity, *n* (%)			
Leukopenia	51 (15.7)	32 (9.9)	0.012
Anemia	23 (7.1)	14 (4.3)	0.14
Thrombocytopenia	41 (12.7)	30 (9.3)	0.32

Acute grade 3 nonhematologic toxicity, *n* (%)			
Nausea-vomiting	109 (33.7)	67 (20.7)	0.006
Esophagitis	49 (15.2)	29 (9.0)	0.003
Pneumonitis	47 (14.5)	27 (8.4)	0.002
Peripheric neuropathy	10 (3.1)	11 (3.4)	0.76
Pericarditis	5 (1.5)	2 (0.6)	0.25

Late grade 3-4 toxicity			
Lung	12 (3.7)	9 (2.8)	0.34
Esophagus	15 (4.6)	8 (2.5)	0.038
Heart	5 (1.5)	3 (0.9)	0.78
Major vessels	3 (0.9)	2 (0.6)	0.64

Late grade 5 toxicity, *n* (%)	6 (1.8)	4 (1.2)	0.73
Radiation pneumonitis	2 (0.6)	1 (0.3)	
Tracheoesophageal fistula	2 (0.6)	1 (0.3)	
Bronchopleural fistula	1 (0.3)	1 (0.3)	
Aortic blow-out	1 (0.3)	1 (0.3)	

Hospitalization, *n* (%)	58 (18.0)	28 (8.6)	<0.001

PFS			
Median, mo	11.7 (10.8–12.6)	11.2 (10.2–12.4)	0.57
5-years (%)	12.1	11.6	
10 years (%)	6.2	5.2	

LRPFS			
Median, mo	15.3 (14.3–16.3)	15.1 (14.1–16.1)	0.52
5-years (%)	13.5	13.3	
10 years (%)	7.2	5.8	

OS			
Median, mo	25.2 (23.5–26.3)	24.6 (22.6)-26.6	0.69
5-year (%)	22.2	21.2	
10 year (%)	11.9	11.1	

5-year in-field failure, *n* (%)			
Local	127 (39.3)	125 (38.7)	0.79
Regional	64 (19.8)	59 (18.3)	0.44
Local + regional	57 (17.6)	61 (18.9)	0.56

Isolated ENF			
5-years (%)	11 (3.4)	14 (4.3)	0.52
10 years (%)	16 (4.9)	19 (5.9)	0.71

ENI: elective nodal irradiation; IFRT: involved-field radiotherapy; PFS: progression-free survival; LRPFS: locoregional progression-free survival; OS: overall survival; ENF: elective nodal failure.

**Table 4:** Results of univariate and multivariate analysis.

Characteristic	Patients (*N*)	Median OS (months)	Univariate *P* value	Multivariate *P* value	Median LRPFS (months)	Univariate *P* value	Multivariate *P* value	Median PFS (months)	Univariate *P* value	Multivariate *P* value
Age group										
<70 years	586	25.8	0.33	—	16.3	0.26	—	12.8	0.19	—
≥70 years	60	22.5			14.1			10.9		

Gender										
Male	400	24.1	0.51	—	14.4	0.62	—	10.9	O.48	—
Female	246	25.5			15.9			12.6		

KPS										
90–100	461	27.9	0.002	0.004	17.6	0.001	0.001	12.8	0.0011	0.024
70–80	185	22.6			13.9			10.7		

Histology										
AC	348	24.4	0.84	—	14.7	0.75	—	12.2	0.39	—
SCC	298	25.3			15.9			11.0		

T-stage										
1-2	289	39.3	<0.001	<0.001	17.3	<0.001	<0.001	12.8	<0.001	<0.001
3-4	357	22.2			13.1			10.6		

N-stage										
2	325	30.4	<0.001	<0.001	19.3	<0.001	<0.001	14.7	<0.001	<0.001
3	321	19.9			11.0			8.3		

Stage										
IIIB	385	33.4	<0.001	<0.001	19.1	<0.001	<0.001	15.4	<0.001	<0.001
IIIC	261	20.2			10.8			7.9		

Chemotherapy cycles										
2-3	529	26.8	<0.001	<0.001	17.3	<0.001	<0.001	12.8	<0.001	<0.001
1	117	20.2			11.4			9.3		

RT group										
ENI	323	25.2	0.69	—	15.3	0.49	—	11.7	0.57	—
IFRT	323	24.6			15.1			11.2		

OS: overall survival; LRPFS: locoregional progression-free survival; PFS: progression-free survival; KPS: Karnofsky performance score; SCC: squamous cell cancer; AC: adenocarcinoma; T: tumor; N: node; RT: radiotherapy; ENI: elective nodal irradiation; IFRT: involved-field radiotherapy.

## Data Availability

The datasets used and/or analyzed during the current study are available from the Baskent University Department of Radiation Oncology Institutional Data Access for researchers who meet the criteria for access to confidential data (contact address: adanabaskent@baskent.edu.tr).
